# Altered FGF Signaling Pathways Impair Cell Proliferation and Elevation of Palate Shelves

**DOI:** 10.1371/journal.pone.0136951

**Published:** 2015-09-02

**Authors:** Weijie Wu, Shuping Gu, Cheng Sun, Wei He, Xiaohua Xie, Xihai Li, Wenduo Ye, Chunlin Qin, Yiping Chen, Jing Xiao, Chao Liu

**Affiliations:** 1 Department of Stomatology, Shanghai Zhongshan Hospital, Shanghai, China; 2 Department of Cell & Molecular Biology, Sciences and Engineering School, Tulane University, New Orleans, Louisiana, United States of America; 3 Department of Biomedical Sciences, Baylor College of Dentistry, Texas A&M Health Sciences Center, Dallas, Texas, United States of America; 4 Department of Oral and Maxillofacial Surgery, Affiliated Stomatological Hospital, Zunyi Medical University, Zunyi, China; 5 Department of Endodontics, Institute of Hard Tissue Development and Regeneration, the 2^nd^ Affiliated Hospital of Harbin Medical University, Harbin, China; 6 Academy of Integrative Medicine, Fujian University of Traditional Chinese Medicine, Fuzhou, China; 7 Department of Oral Biology, College of Stomatology, Dalian Medical University, Dalian, China; Columbia University, UNITED STATES

## Abstract

In palatogenesis, palatal shelves are patterned along the mediolateral axis as well as the anteroposterior axis before the onset of palatal fusion. *Fgf10* specifically expressed in lateral mesenchyme of palate maintains *Shh* transcription in lateral epithelium, while *Fgf7* activated in medial mesenchyme by *Dlx5*, suppressed the expansion of *Shh* expression to medial epithelium. How FGF signaling pathways regulate the cell behaviors of developing palate remains elusive. In our study, we found that when *Fgf8* is ectopically expressed in the embryonic palatal mesenchyme, the elevation of palatal shelves is impaired and the posterior palatal shelves are enlarged, especially in the medial side. The palatal deformity results from the drastic increase of cell proliferation in posterior mesenchyme and decrease of cell proliferation in epithelium. The expression of mesenchymal *Fgf10* and epithelial *Shh* in the lateral palate, as well as the *Dlx5* and *Fgf7* transcription in the medial mesenchyme are all interrupted, indicating that the epithelial-mesenchymal interactions during palatogenesis are disrupted by the ectopic activation of mesenchymal *Fgf8*. Besides the altered *Fgf7*, *Fgf10*, *Dlx5* and *Shh* expression pattern, the reduced *Osr2* expression domain in the lateral mesenchyme also suggests an impaired mediolateral patterning of posterior palate. Moreover, the ectopic *Fgf8* expression up-regulates pJak1 throughout the palatal mesenchyme and pErk in the medial mesenchyme, but down-regulates pJak2 in the epithelium, suggesting that during normal palatogenesis, the medial mesenchymal cell proliferation is stimulated by FGF/Erk pathway, while the epithelial cell proliferation is maintained through FGF/Jak2 pathway.

## Introduction

Mammalian palate is constituted by hard and soft palates. The hard palate originates in a small extent from the primary palate and dominantly from secondary palate, while the soft palate is entirely contributed by the secondary palate. Both the palatal primordia, especially the secondary palate are required for the separation of oral cavity to form nasal airway, which is essential for normal feeding and speaking. The studies on mammalian palatogenesis by using mouse models reveal that the secondary palate protrudes out of the maxillary prominences at embryonic day 11.5 (E11.5). In the following E12.5–13.5, the bilateral palatal shelves extend downward beside the developing tongue. At the E14.0, the palatal shelves elevate horizontally and fuse with each other to form an intact palate roof [1]. The growth, elevation and fusion of palatal shelves are accomplished through a series of cell behaviors, including cell proliferation, apoptosis, migration and epithelial-mesenchymal transition (EMT) [2, 3, 4]. Any perturbation in these behaviors will lead to cleft palate, making palatogenesis susceptible to the genetic and environmental influence and cleft palate one of the most common congenital birth defects in human population.

The development of secondary palate is accomplished by the reciprocal interactions between the neural crest-derived mesenchyme and the overlying pharyngeal ectoderm. The transcription of *Sonic hedgehog (Shh)* is first detected in the earliest epithelial ruga, which defines the boundary between the hard and soft palates at the E12.0 [5]. During palatogenesis, *Shh* transcription is restricted to the stripes of rugae [5, 6]. SHH is regarded as a central signal molecule integrating Fibroblast Growth Factor (FGF) and Bone Morphogenic Protein (BMP) signaling during palate development [1, 5, 6, 7]. Epithelial SHH stimulates cell proliferation in the underlying palatal mesenchyme and the expression of *Fgf10* through SHH/Smo signaling [7]. The mesenchymal FGF10 in turn maintains the expression of *Shh* and cell proliferation in palatal epithelium by activating *Fibroblast Growth Factor Receptor 2b (FGFR2b)* [7, 8, 9]. Also through the SHH/Smo signaling, SHH maintains the expression of *Bmp2* in the palatal epithelium, but weakens *Bmp4-Msx1*feedback loop in the mesenchyme [7].

Although homogeneous in appearance, the palatal shelf displays the heterogeneity of gene expression along the anteroposterior (A-P) and mediolateral (M-L) axes. The expression of *Bmp2*, *Bmp4*, *Wnt5a* and its receptor, *Ror2*, are robust in the anterior half and decrease gradually along A-P axis [4, 10]. Correspondingly, the transcription factors, *Msh homeobox1 (Msx1)* and *Short Stature homeobox 2 (Shox2)* are predominantly expressed in the anterior palate, but absent from the posterior palate [10, 11]. In contrast, the *BarH-like homebox 1 (Barx1)*, *Meningioma 1 (Mn1)*, *Mesenchymal homeobox 2 (Meox2)*, and *T-box transcription factor 22 (Tbx22)* are only activated in the posterior palatal mesenchyme [1, 5, 12, 13, 14]. Similar to the differential expression pattern along A-P axis, *Odd-skipped related-2 (Osr2)* and *Wnt5a* also exhibit a gradient expression pattern along the M-L axis of the palatal shelf [4, 15]. Most intriguingly, *Distal-less homeobox 5 (Dlx5)* activated in the medial mesenchyme is able to suppress *Shh* transcription in the medial epithelium of palate by activating *Fgf7* expression [16]. Since *Fgf10* and *Fgf7* are activated in the lateral mesenchyme adjacent to the *Shh*-expressing rugae and the medial mesenchyme, respectively, it implicates that *Shh-Fgf10* and *Dlx5-Fgf7* feedback loops may be associated with the establishment of the M-L polarity of palatal shelf [1, 16].

Binding of FGF ligands to FGFRs triggers the phosphorylation of tyrosine residues in the cytoplasmic domain of FGFRs. The activated FGFRs transduce the signals through four intracellular pathways: phosphatidylinositol 3-kinase/Akt (PI3K/Akt), Janus kinase/signal transducer and activator of transcription (Jak/Stat), phosphoinositide phospholipase C (PLCγ) and Erk pathways [17]. The intracellular pathways activated by a certain FGF ligand largely depend on the extra- and intracellular context [17, 18, 19]. Although the roles of FGF7 and FGF10 in palate development have been reported, their exact intracellular mechanism pathways remain elusive. In our study, we conditionally activated *Fgf8* in the developing palatal mesenchyme by using *Rosa26R-Fgf8* and *Osr2-cre* ^*KI*^ alleles [20, 21]. We found that the ectopically expressed *Fgf8* disturbed the expression of *Fgf7* and *Fgf10*, which provided us a useful tool to explore the role of FGF signaling in the palate development.

## Materials and Methods

### Ethics Statement

All animals used in this study were bred and treated in strict accordance with the recommendations in the Guide for the Care and Use of Laboratory Animals of the National Institutes of Health and in compliance with animal protocol approved by the Committee on the Ethics of Animal Experiments at Tulane University (Protocol Number: 0184-R4).

### Mouse lines

The *Osr2-cre* ^*KI*^ mouse was obtained from Dr. Jiang’s lab at Cincinnati Children’s Hospital Medical Center [20]. The *Rosa26R-Fgf8* line, generated by Dr. Ma at Washington University School of Medicine, has been described previously [21]. We crossed the *Osr2-cre* ^*KI*^ mouse with *Rosa26R-Fgf8* mouse to get the E12.5–18.5 *Osr2-cre* ^*KI*^;*Rosa26R-Fgf8* mutants. The *K14-cre* and *Shh-cre* lines, previously applied in craniofacial research, were purchased from Jackson Laboratories [22, 23]. For the analysis of Cre activity, the *Osr2-cre* ^*KI*^ or *Shh-cre* mouse was mated with *Rosa26R-LacZ* mouse to get the embryos from E11.5-E13.5 [24]. The mated mice were checked for vaginal plugs every morning and the noon of plug day was recorded as 0.5 day of pregnancy (E0.5).

### X-gal staining and histological analysis

The E11.5-E13.5 *Osr2-cre* ^*KI*^;*Rosa26R-Fgf8* embryos were fixed in 0.25% glutaraldehydeat 4°C overnight, passed through 15% and 30% sucrose series, embedded in O.C.T. Compound, and cryo-sectioned at 10 μm. X-gal staining for cryostat sections were performed as described in standard protocol [25]. The E11.5-E18.5 mouse heads were fixed in 4% paraformaldehyde (PFA)/PBS, dehydrated through graded ethanol, cleared with xylene, embedded in paraffin, and then, sectioned at 10 μm for standard Hematoxylin/Eosin staining [26]. At least three samples were examined at each stage.

### Cell proliferation assay

Cell proliferation in the E13.5 embryos was performed by the detection of incorporated BrdU on 10 μm paraffin-embedded sections as described in the previous report [27]. The proliferation index of palatal mesenchyme was defined as the number of BrdU-positive nuclei in a fixed area of the medial or lateral palatal shelves, while the proliferation index of palatal epithelium was calculated as the ratio of the BrdU positive nuclei in 100 counted nuclei. Three samples and at least four sections of each samples were counted and statistically analyzed by student t-test.

### *In situ* hybridization analysis

For section *in situ* hybridization, samples were harvested in ice-cold PBS and fixed in 4% PFA/PBS at 4°C overnight prior to dehydration through graded ethanol and embedding in paraffin. Samples were sectioned at 10 μm and subjected to non-radioactive *in situ* hybridization as described previously [11]. The validity of the anti-sense probes for *Dlx5*, *Fgf7*, *Fgf10*, *Osr2* and *Wnt5a* has been reported previously [4, 7, 16]. The whole mount *in situ* hybridization with anti-sense *Shh* probe was performed as described previously [28]. Both the section and whole mount *in situ* hybridization were repeated in the samples collected from three litters.

### Immunohistochemistry

For immunohistochemical staining, samples were fixed in Z-fix (Anatech) at room temperature for 2 hours, dehydrated with gradient alcohol, cleared with xylene, embedded in paraffin, and sectioned at 10 μm. The rabbit anti-mouse polyclonal antibodies against pAkt, pErk1/2, pJak1, pJak2 and pPLCγ1 were all produced by Santa Cruz Biotechnology, Inc. The horseradish peroxidase-coupled goat anti-rabbit IgG was purchased from Vector Laboratories, Inc. Immunohistochemical staining was conducted according to the manufacturer’s instruction using above antibodies. Each antibody was applied on at least three sections from different samples.

## Results

### *Cre* activity was specifically distributed in the palatal mesenchyme of *Osr2-cre* ^*KI*^ mouse

**Fig 1:**
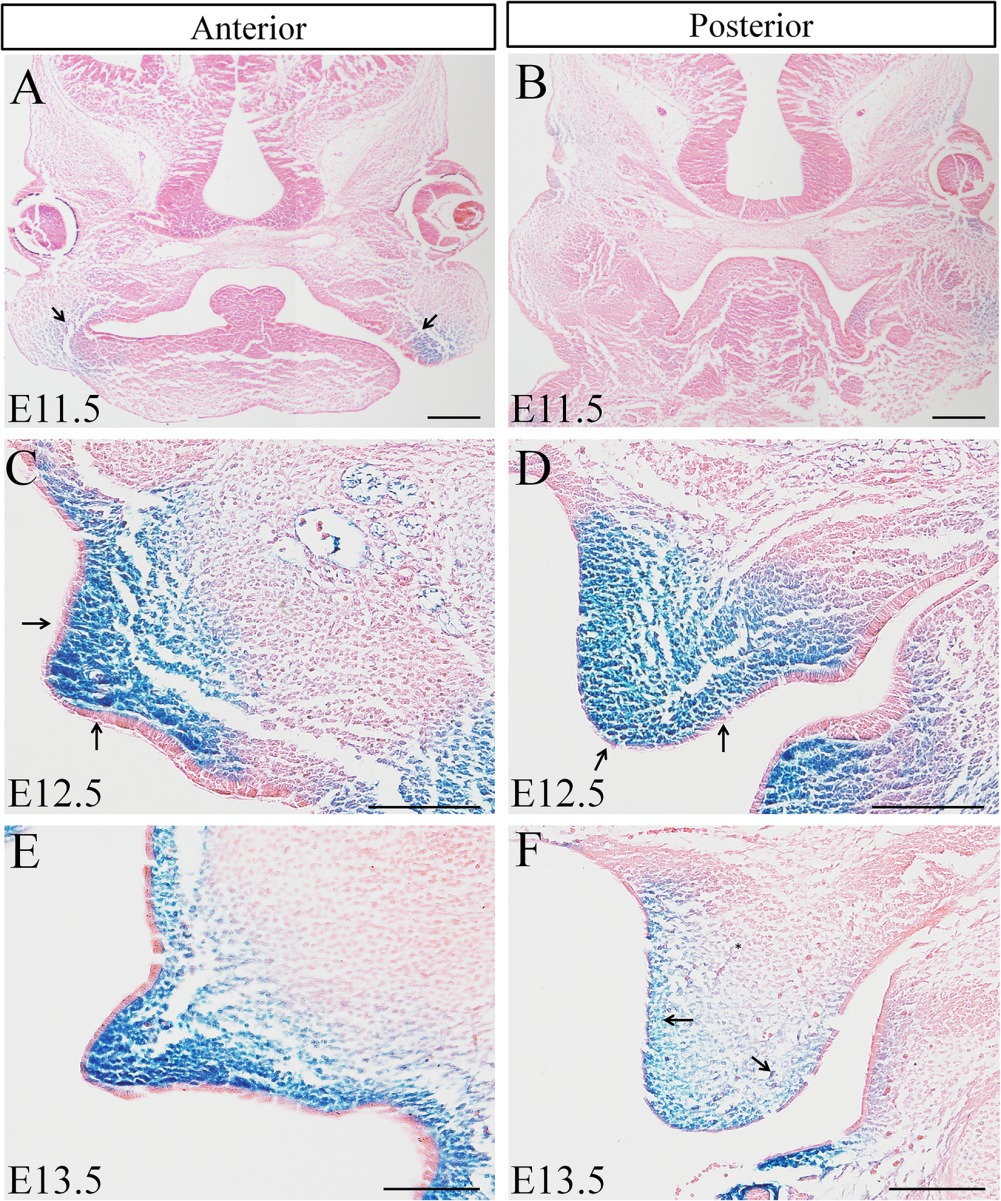
*Cre* pattern in the developing palatal shelves detected by *lacZ* expression in *Osr2-Cre* ^*KI*^ *; Rosa26R-LacZ* embryos. (A-F) *Cre*-mediated *LacZ* expression in the developing *Osr2-Cre* ^*KI*^ *; Rosa26R-LacZ* palatal shelves. At E11.5, both the nascent anterior (A) and posterior palatal shelves (B) were devoid of *LacZ* staining. A slight *LacZ* staining was only detected in the connecting area between mandible and maxillary process (Arrows in A). At E12.5, *Cre*-mediated *LacZ* expression was activated throughout the anterior (C) and posterior palatal mesenchyme (D), but specifically absent from the palatal epithelium (arrows in C&D). At E13.5, Cre activity evenly distributed anterior palate mesenchyme (E). However, Cre activity was only found in the medial and lateral mesenchyme of the posterior palate (arrowheads in F), but absent from the central part (_*_ in F). Judged by the *LacZ* intensity, there was no difference in *Cre* activity between the medial and lateral palatal mesenchyme (C-F). (Scale bar: 200um)

Previous studies have reported that the expression of *Osr2* gene in the developing palate was specifically restricted in the mesenchyme with an increasing gradient from medial to lateral side [15, 29, 30]. The *Cre* following IRES sequence targeted into the 3’-untranslated region (UTR) of *Osr2* locus (*Osr2* ^*IresCre*^) has been applied for the study of mouse palatal development as a valid tool [7, 31]. However, the *Cre* activity of *Osr2* ^*IresCre*^ mouse was detected all-over the palatal mesenchyme from E11.5 without a medial-lateral gradient [29, 32]. Moreover, a subset of *Osr2* ^*IresCre*^ mouse embryos exhibited an ectopic *Cre* activity, which might affect the accuracy of the interpretation of phenotypes [29]. To circumvent the influence of the ectopic *Cre* activity, we adopted the *Osr2-cre* ^*KI*^ line replacing the *Osr2* coding sequence with *Cre* cDNA in our following study [20]. When we crossed the *Osr2-cre* ^*KI*^ line with the *Rosa26R-LacZ* reporter line, *Cre*-mediated activation of *LacZ* expression showed that at E11.5, the *Cre* activity only could be detected in the mesenchyme connecting the mandible and maxillary processes, but excluded from anterior and posterior palate shelves (Fig 1A and 1B). In the E12.5 palatal shelves, *Cre* activity was distributed throughout the anterior and posterior mesenchyme without a medial to lateral gradient (Fig 1C and 1D). In the E13.5 palatal shelves, although the *LacZ* pattern showed an all-over distribution of *Cre* activity in the anterior palatal mesenchyme, but in the posterior palate, *Cre* activity was only detected in the medial and lateral mesenchyme with the same intensity, and absent from the central mesenchyme (Fig 1E and 1F).

### Over-expression of *Fgf8* by *Osr2-Cre* ^*KI*^ results in complete cleft palate

**Fig 2:**
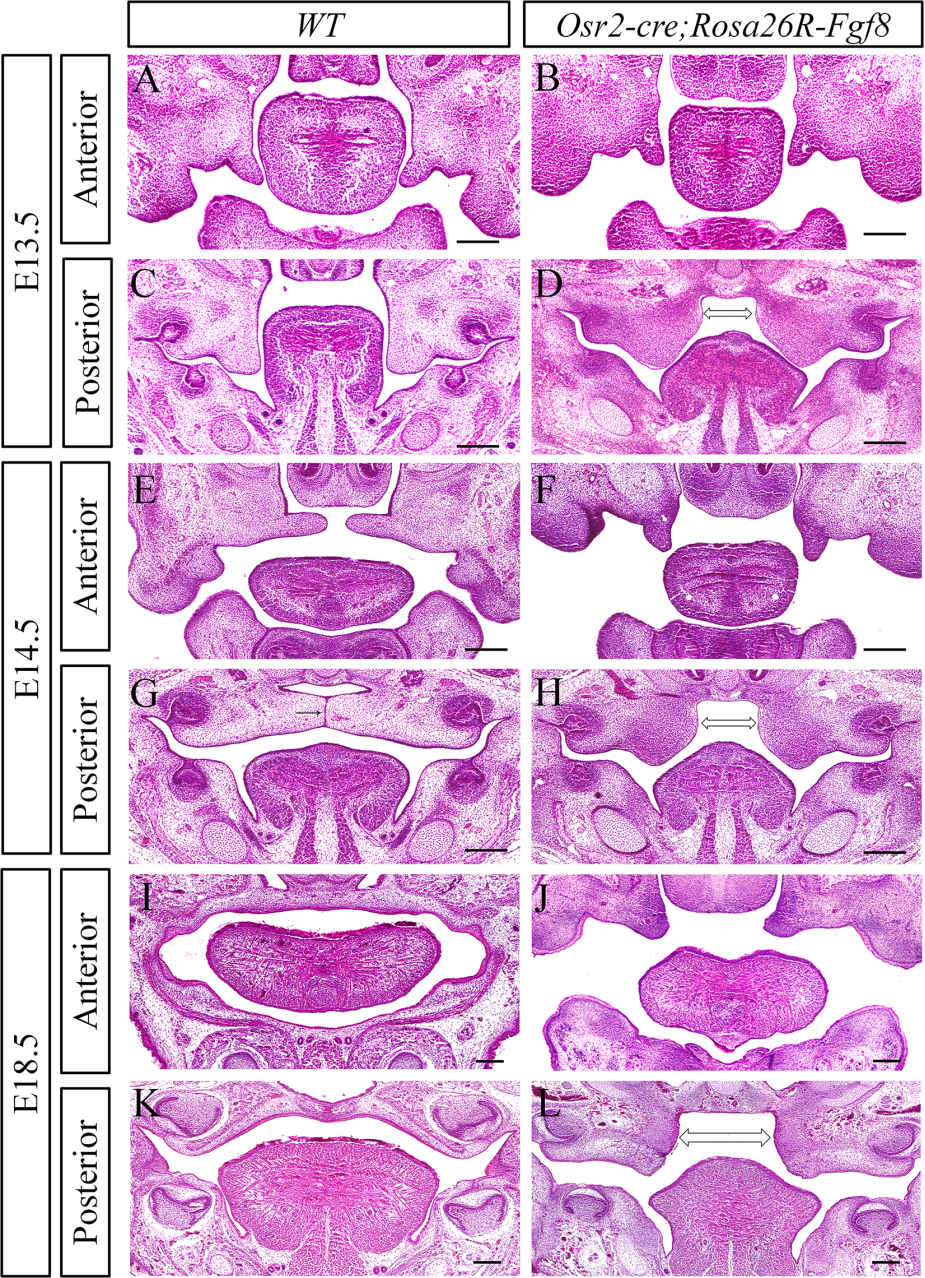
The complete cleft palate in *Osr2-Cre* ^*KI*^ *; Rosa26R-Fgf8* mouse embryos. (**A-L**) Cross sections by HE staining of the developing *Osr2-Cre* ^*KI*^ *; Rosa26R-Fgf8* palatal shelves. At E13.5, the anterior palatal shelves showed no difference between the WT (A) and *Osr2-Cre* ^*KI*^ *; Rosa26R-Fgf8* (B) embryos. By contrast, the mutant posterior palatal shelves (D) were significantly enlarged in size, compared with the WT posterior palatal shelves (C), especially in the medial side (bidirectional arrow in D). When the WT palatal shelves elevated horizontally at the anterior (E) and even initiated fusion by forming middle epithelial seam in the posterior (arrow in G) at E14.5, the *Osr2-Cre* ^*KI*^ *; Rosa26R-Fgf8* palatal shelves still kept their vertical status in both anterior (F) and the posterior (H) with the obvious enlarged medial side (bidirectional arrow in H). At E18.5, the *Osr2-Cre* ^*KI*^ *; Rosa26R-Fgf8* embryos exhibited the complete cleft palate: the anterior palatal shelves only elevated slightly (J) and the enlarged posterior shelves showed no sign of elevation (L, bidirectional arrow delineated the enlarged medial mesenchyme). By contrast, the E18.5 WT palate shelves had fused with each other and separated oral from nasal cavity in the anterior (I) and posterior (K) (Scale bar: 200um)

To investigate the influence of over-dosage FGF8 on the palate development, we crossed the *Osr2-Cre* ^*KI*^ line with the *Rosa26R-Fgf8* line [21]. The *Osr2-Cre* ^*KI*^ *;Rosa26R-Fgf8* embryos displayed normal palatal outgrowth up to E12.5 (data not shown). At E13.5, the anterior palatal shelves of *Osr2-Cre* ^*KI*^; *Rosa26R-Fgf8* embryos were comparable to the wild type littermates (Fig 2A and 2B), but the posterior palatal shelves were enlarged, especially in the medial side (Fig 2C and 2D). At E14.5, the anterior palatal shelves had elevated to horizontal position in the wild type embryos, while still being kept in the vertical position in the mutant littermates (Fig 2E and 2F). The E14.5 wild type posterior palatal shelves had contacted with each other and formed midline epithelial seam after elevation (Fig 2G). Whereas, the posterior shelves of the E14.5 *Osr2-Cre* ^*KI*^ *;Rosa26R-Fgf8* embryo failed to elevate (Fig 2H). The secondary palate of wild type embryos completely fused and separated oral from nasal cavity in both the anterior and posterior halves at E18.5 (Fig 2I and 2K), but the enlarged mutant palatal shelves only elevated slightly in the anterior, and stayed vertical in the posterior half (Fig 2J and 2L), resulting in a complete cleft palate which was lethal to the newborn mice.

### Abnormal cell proliferation in *Osr2-Cre* ^*KI*^; *Rosa26R-Fgf8* palatal shelves

**Fig 3:**
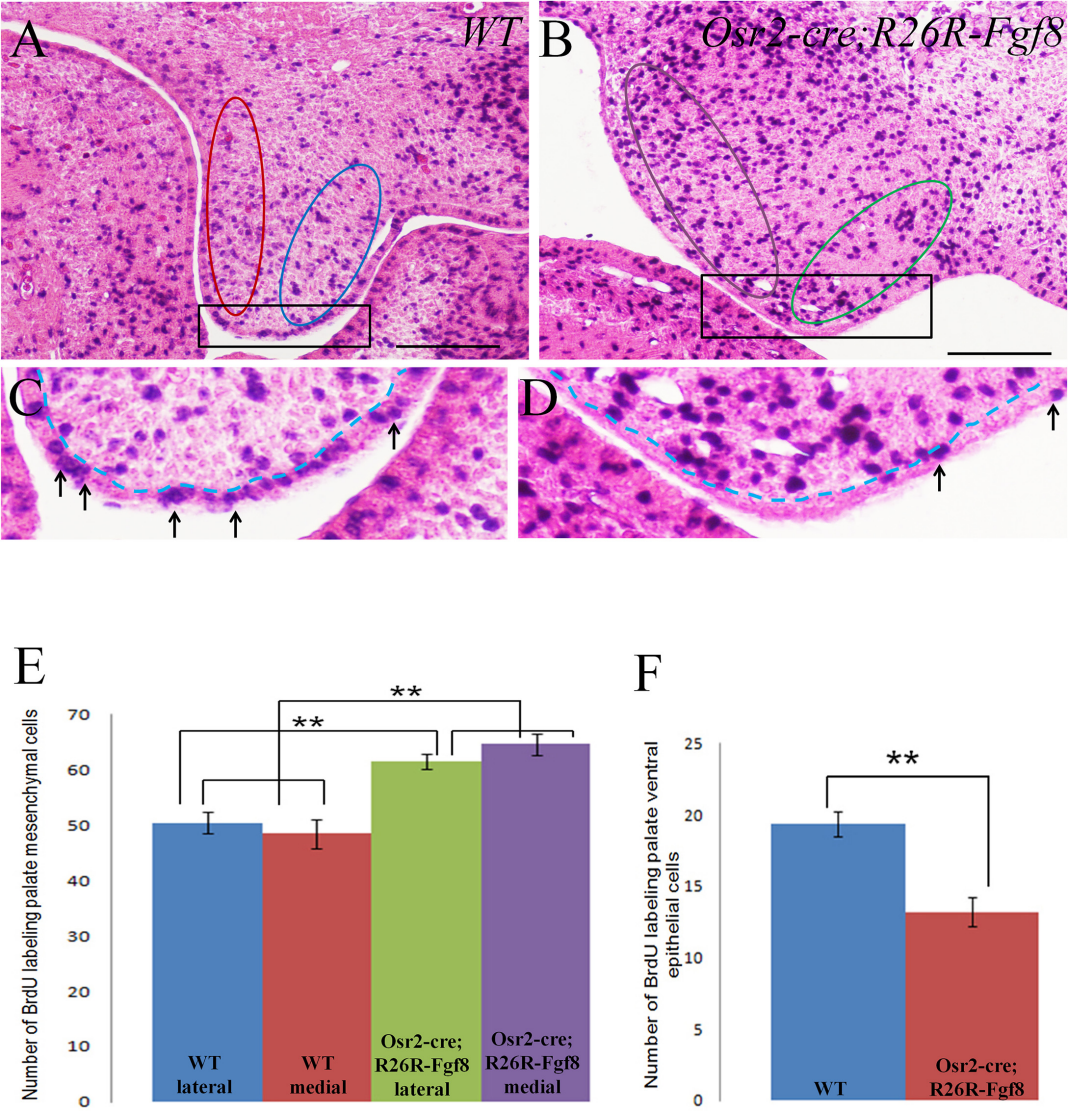
The examination on cell proliferation ratio of the developing palatal shelves of *Osr2-Cre* ^*KI*^ *; Rosa26R-Fgf8* mouse embryos. (**A-D**) Cell proliferation test by BrdU labelling for the E13.5 *Osr2-Cre* ^*KI*^ *; Ros26R-Fgf8* palatal shelves. The BrdU labelled cells in the E13.5 WT medial (red circle in A) and lateral palatal mesenchyme (blue circle in A) were less than those in the *Osr2-Cre* ^*KI*^ *; Ros26R-Fgf8* medial (violet circle in B) and lateral mesenchyme (green circle in B). Opposing to the mesenchymal cell proliferation ratio, the number of BrdU positive cells in the E13.5 WT palatal epithelium (arrows in C) was more than that in the mutant palatal epithelium (arrows in D). (**E, F**) Statistics of the numbers of BrdU labelled cells in the E13.5 palatal shelves. The number of proliferating medial mesenchymal cells significantly raised from 48.455 (SD = 8.722, SE = 2.629) in WT to 64.667 (SD = 5.937, SE = 1.979) in the *Osr2-Cre* ^*KI*^ *; Rosa26R-Fgf8* mouse (P<0.01). Similarly, the proliferating cells in lateral mesenchyme raised greatly from 50.455 (SD = 6.758, SE = 1.983) in WT to 61.556 (SD = 4.362, SE = 1.454) in the *Osr2-Cre* ^*KI*^ *; Rosa26R-Fgf8* mouse (P<0.01) (E). The difference in cell proliferation ratio between the medial and lateral palatal mesenchyme had no significance in both WT and *Osr2-Cre* ^*KI*^ *; Rosa26R-Fgf8* mouse (E). Reversely, the amount of proliferating cells in palatal epithelium dropped drastically from 19.364 (SD = 2.942, SE = 0.887) in WT to 13.322 (SD = 3.114, SE = 1.038) in the *Osr2-Cre* ^*KI*^ *; Ros26R-Fgf8* mouse (P<0.01) (F). (C and D were the enlarged areas of the square boxes in A and B, respectively; Dashed lines in C and D delineated the boundary between mesenchyme and epithelium; SD, Standard Derivation; SE, Standard Error; Scale bar: 200um)

To explore how the constitutively activated *Fgf8* enlarged the posterior palatal shelves, we compared the cell proliferation by BrdU labelling test between wild type and *Osr2-Cre* ^*KI*^; *Rosa26R-Fgf8* posterior palatal shelves. Compared with their wild type littermates, the amount of BrdU labelled cells of E13.5 *Osr2-Cre* ^*KI*^; *Rosa26R-Fgf8* posterior palatal shelves increased significantly in both medial and lateral mesenchyme (Fig 3A, 3B and 3E), but decreased drastically in the epithelium (from 19.364 to 13.222; Fig 3C, 3D and 3F). The number of BrdU positive cells of the medial mesenchyme (64.667) was higher than that of the lateral mesenchyme (61.556) in the E13.5 *Osr2-Cre* ^*KI*^; *Rosa26R-Fgf8* palatal shelves (Fig 3E). In contrast, the proliferating medial mesenchymal cells (48.455) were less than those of lateral side (50.455) in the wild type palatal shelves (Fig 3E). However, these differences had no statistical significance (Fig 3E). The BrdU-labelling examination indicated that the enlarged palatal shelves of *Osr2-Cre* ^*KI*^; *Rosa26R-Fgf8* mice resulted from the increased mesenchymal cell proliferation stimulated by the mesenchyme derived FGF8. It also implied that the palatal mesenchyme derived FGF8 had an inhibitory effect on the cell proliferation of palatal epithelium.

### FGF8 had no direct effects on the *Shh* expression and cell proliferation in palatal epithelium

**Fig 4:**
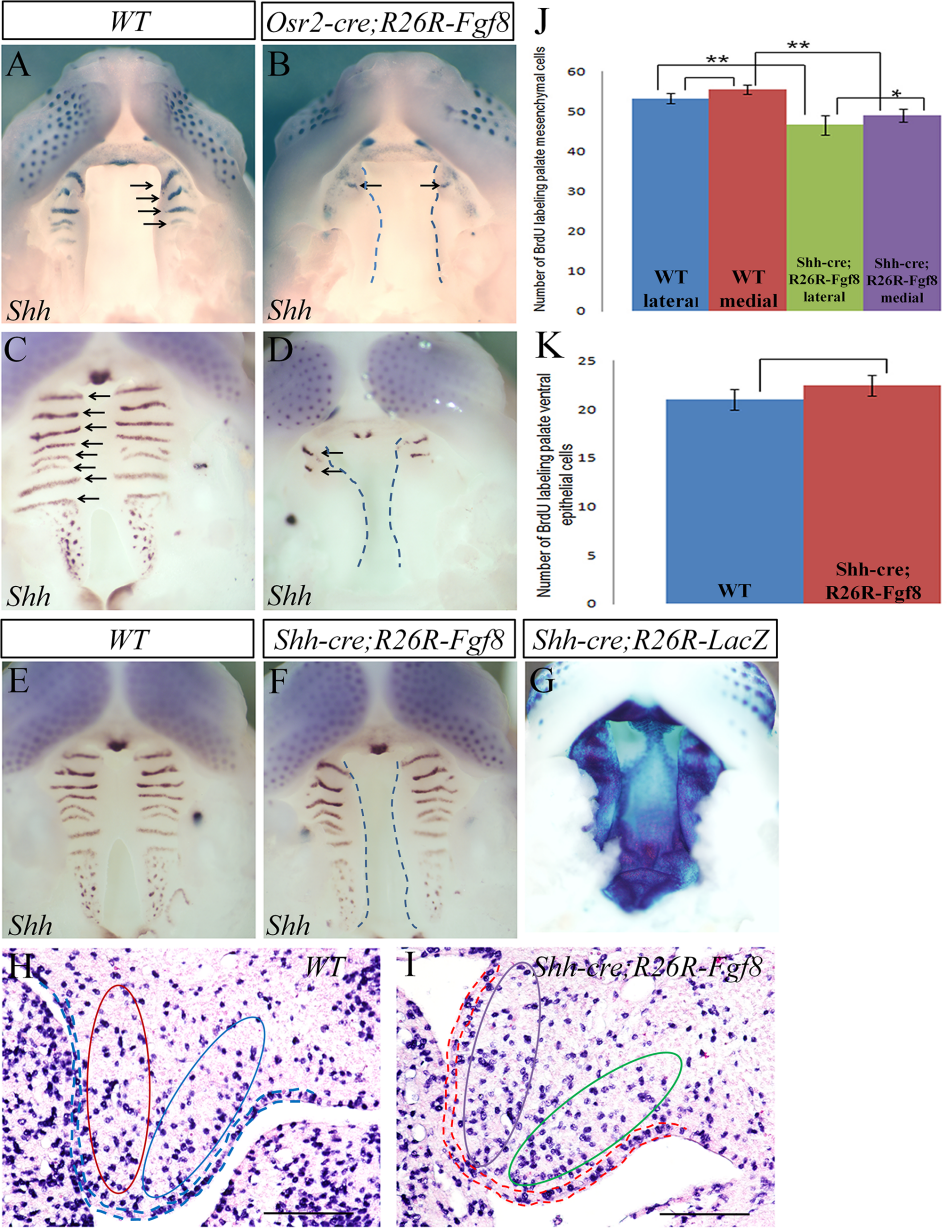
FGF8 does not directly inhibit *Shh* expression and palatal epithelial cell proliferation. **(A-D)** Whole mount *in situ* hybridization for *Shh* expression in *Osr2-Cre* ^*KI*^ *;Rosa26R-Fgf8* palate. At E13.5, a robust *Shh* transcription was detected in the most anterior rugae of the WT palate (arrows in A); only a trace of *Shh* transcription was found in the most anterior ruga in mutant littermate (arrow in B). At E14.5, there were 8 *Shh*-expressing rugae in WT palate (arrows in C), while in *Osr2-Cre* ^*KI*^ *;Rosa26R-Fgf8* palate, only two short *Shh*-expressing rugae were emerging (arrows in D). (**E, F**) Whole mount *in situ* hybridization with *Shh* probe in *Shh-Cre;Rosa26R-Fgf8* palate. Although a complete cleft palate was detected in *Shh-Cre;Rosa26R-Fgf8* mouse at E14.5 (F), both the WT (E) and their mutant littermates (F) show 8 *Shh*-expressing rugae. (**G**) X-gal staining demonstrates that at E13.5, Cre activity was distributed all over the palatal epithelium of the *Shh-Cre;Rosa26R-LacZ* mouse. (**H-K**) BrdU labelled test and statistical analysis for E13.5 palatal cell proliferation. The E13.5 cell proliferation of WT (H) and *Shh-Cre;Rosa26R-Fgf8* (I) palatal shelves showed significant difference in mesenchyme (J) and no difference in epithelium (K). The BrdU labelled cells of *Shh-Cre;Rosa26R-Fgf8* lateral mesenchyme (42.625, SD = 6.805, SE = 2.404; violet column in J) were significantly less than that of WT control (53.25, SD = 3.615, SE = 1.278; red column in J) (P<0.01); similarly, the BrdU positive cells in medial *Shh-Cre;Rosa26R-Fgf8* mesenchyme (49, SD = 4.44, SE = 1.569; green column in J) were dramatically less than WT littermate (55.625, SD = 3.335, SE = 1.179; blue column in J) (P<0.01). Although the cell proliferation of both the medial and lateral *Shh-Cre;Rosa26R-Fgf8* mesenchyme decreased, the drop in the lateral side was more significant than the medial side (P<0.05). The BrdU labelled epithelial cells in *Shh-Cre;Rosa26R-Fgf8* palate (22.5, SD = 2.976, SE = 1.052; red column in K) were slightly more than WT control (21, SD = 3.023,SE = 1.069; blue column in K), but the increase has no significance (P>0.05) (Red and blue circles in H and violet and green circles in I delineate medial and lateral mesenchyme, respectively; Dashed lines in B, D and F delineate the edge of palatal shelves; Dashed lines in H and I stand for the layer of epithelium; SD, Standard Deviation;SE, Standard Error; Scale bar: 200um)

SHH directly stimulates the mesenchymal cell proliferation of the developing palatal shelves and indirectly promotes epithelial cell proliferation by activating mesenchymal *Fgf10* [5, 7]. Whole mount *in situ* hybridization with *Shh* probe was performed to examine if FGF8 up-regulated the *Shh* transcription to stimulate mesenchymal cell proliferation. Surprisingly, when *Shh* was activated in the most anterior 3 rugae of the WT palatal shelves at E13.5, only a slight expression of *Shh* was detected in the most anterior ruga of the *Osr2-Cre* ^*KI*^ *; Rosa26R-Fgf8* palatal shelves (Fig 4A and 4B). At E14.5, when *Shh* expression was detected in all 8 rugae of the fused wild type palate, there were only two short *Shh*-expressing rugae found in the mutant littermates (Fig 4C and 4D). These phenomena suggested that the epithelial *Shh* transcription was inhibited by ectopic FGF8. To examine if FGF8 exerted a direct inhibition on *Shh* transcription, the *Rosa26R-Fgf8* allele was constitutively activated by *K14-Cre* and *Shh-Cre*. Intriguingly, cleft palate was only detected in the *Shh-Cre;Rosa26R-Fgf8*embryos with an unaffected *Shh* expression in palatal shelves (Fig 4E, 4F and 4G), and not detected in the *K14-Cre;Rosa26R-Fgf8* mice at all (data not shown). Furthermore, although the cell proliferation exhibited a marked decrease in both the E13.5 medial and lateral *Shh-Cre;Rosa26R-Fgf8* palatal mesenchyme compared with their wild type controls (Fig 4H and 4J), there was no significant difference in the palatal epithelia between the E13.5 WT and *Shh-Cre;Rosa26R-Fgf8* littermates (Fig 4H, 4I and 4K). Therefore, FGF8 does not directly suppress *Shh* expression and cell proliferation in palatal epithelium. Taken together, the disrupted *Shh* expression in the *Osr2-Cre* ^*KI*^ *;Rosa26R-Fgf8* palatal epithelium was supposed to be attributed to altered gene expression in palatal mesenchyme, instead of the direct inhibition by FGF8.

### The altered gene expression in the *Osr2-Cre* ^*KI*^ *;Rosa26R-Fgf8* palatal mesenchyme

**Fig 5:**
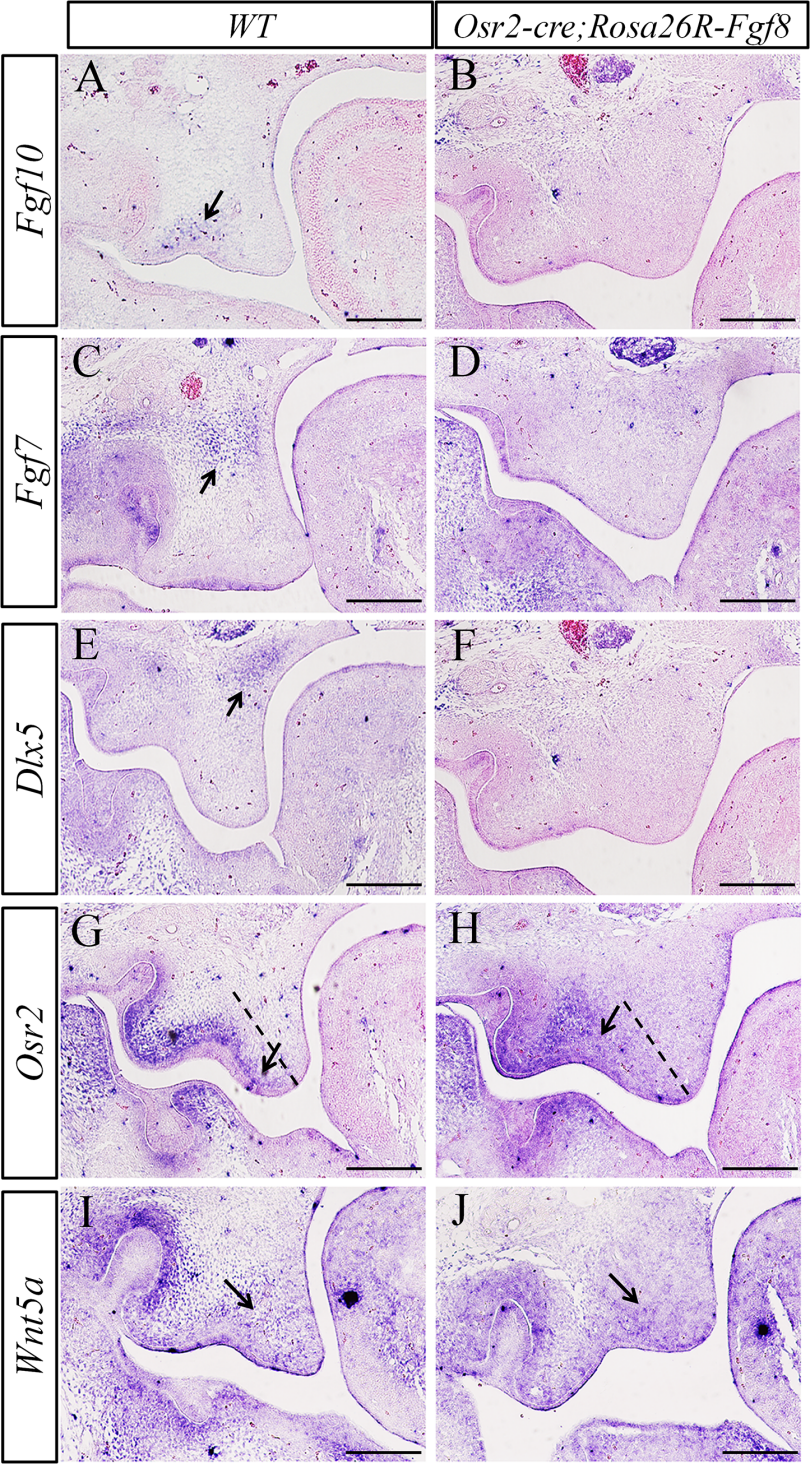
The expression pattern ofmesenchyme markers inthe *Osr2-Cre* ^*KI*^ *;Rosa26R-Fgf8* palate. **(A-J)** *In situ* hybridization for mesenchymal markers of E13.5 palatal shelves. *Fgf10* was activated in the lateral mesenchyme of WT palate (arrow in A), but silenced in *Osr2-Cre* ^*KI*^ *;Rosa26R-Fgf8* palate (B). Similarly, *Fgf7* activated in WT medial palatal mesenchyme (arrow in C) disappeared in mutant palate (D). The expression of *Dlx5* acting as the activator of *Fgf7* was detected in medial side of WT palatal mesenchyme (arrow in E), but not in the palate of mutant littermate (F). Compared with the *Osr2* expression in WT lateral palate (arrow in G), the domain of *Osr2* in the *Osr2-Cre* ^*KI*^ *;Rosa26R-Fgf8* palate reduced from the distal mesenchyme (arrow in H). The expression of *Wnt5a* detected in lateral mesenchyme of the *Osr2-Cre* ^*KI*^ *;Rosa26R-Fgf8* palate (arrow in J) was comparable to that in the WT (arrow in I).

Then, we examined the transcription of a series of mesenchymal markers by *in situ* hybridization. Consistent with the loss of *Shh* expression in epithelial rugae, the expression of *Fgf10* diminished from the lateral mesenchyme in the E13.5 *Osr2-Cre* ^*KI*^ *;Rosa26R-Fgf8* palatal shelves (Fig 5A and 5B), indicating that the increased mesenchymal cell proliferation was attributed to FGF8, instead of FGF10 as in normal palatal development. Identical to medial *Fgf10* expression, the expression of *Fgf7* disappeared in the E13.5 mutant medial palatal shelves (Fig 5C and 5D). As the upstream activator of *Fgf7*, the transcription of *Dlx5* was suppressed in the E13.5 *Osr2-Cre* ^*KI*^ *;Rosa26R-Fgf8* medial palatal mesenchyme (Fig 5E and 5F). Another transcription factor, *Osr2* which was reported to be dominantly expressed in the lateral mesenchyme [15, 33], displayed a reduced expression domain along the *Osr2-Cre* ^*KI*^ *;Rosa26R-Fgf8* lateral palatal mesenchyme (Fig 5G and 5H). However, *Wnt5a*, a growth factor which was also expressed with a priority in lateral palate [4, 34], showed no difference between wild type and mutant palatal shelves (Fig 5I and 5L). These results indicated that the ectopically activated *Fgf8* expression disrupted the *Shh-Fgf10* feedback loop required for cell proliferation in normal palate development, and stimulated the proliferation of medial and lateral palatal mesenchymal cells indiscriminately.

### The altered FGF signaling pathways inthe *Osr2-Cre* ^*KI*^ *;Rosa26R-Fgf8* palatal shelves

**Fig 6:**
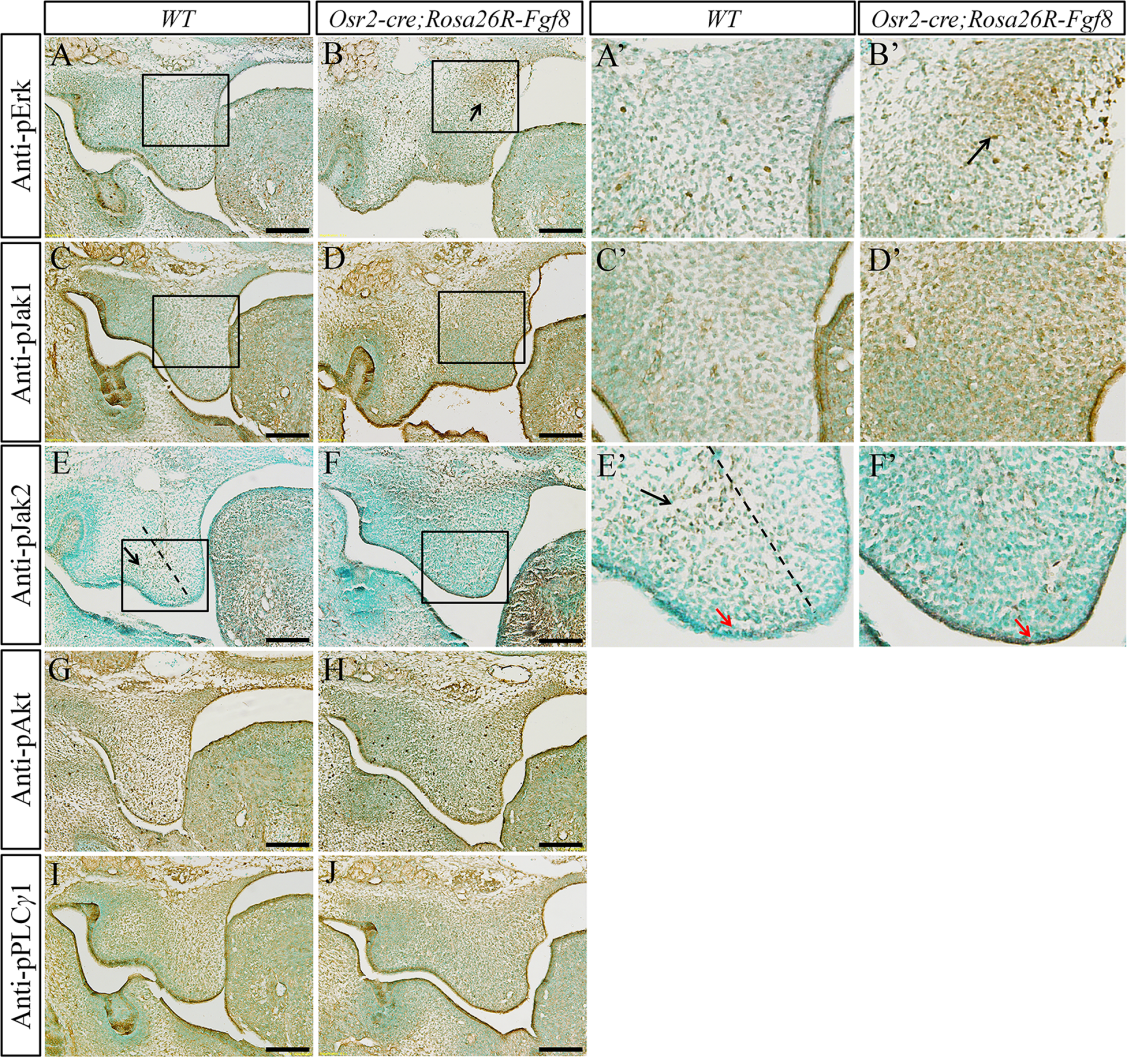
The pattern ofphosphorylated FGF signaling mediators in the *Osr2-Cre* ^*KI*^ *;Rosa26R-Fgf8* palate. **(A-H)** Immunohistochemistry for phosphorylated FGF signaling mediators in E13.5 palatal shelves. pErk was specifically activated in the medial mesenchyme of *Osr2-Cre* ^*KI*^ *; Rosa26R-Fgf8* palate (arrow in B), but absent from WT palate mesenchyme (A). WT palatal mesenchyme was devoid of pJak1 (C), while the mutant palatal mesenchyme showed a strong level of pJak1 (D). Normally, the pJak2 was limited to the WT lateral mesenchyme (arrow in E) and absent from palatal epithelium (E); by contrast, the pJak2 was diminished in *Osr2-Cre* ^*KI*^ *; Rosa26R-Fgf8* palatal mesenchyme and activated in the epithelium (F). The level of pAkt in the mesenchyme and epithelium of WT palate (G) is comparable to that in mutant palate (H). Similarly, compared with the WT control (I), the level of pPLCγ1 showed no difference in *Osr2-Cre* ^*KI*^ *; Rosa26R-Fgf8* palatal mesenchyme and epithelium (J). (**A’-F’**) The enlarged views of the boxed areas in A-F. Black arrow in B’ meant the enhanced pErk area; black arrow in E’ pointed to the active pJak2 region, and red arrows in E’ and F’ to palatal epithelium; the dashed line in E and E’ delineated the boundary between medial and lateral mesenchyme. (Scale bar: 200um)

To reveal how the ectopic FGF8 exerted the function on the developing palatal mesenchyme, antibodies against pivotal transducers of FGF signaling pathways were applied in immunohistochemistry. There are four intracellular pathways mediating the biological function of FGF ligands, including PI3K/Akt pathway, Erk pathway, Jak/Stat pathway and PLCγ pathway [17]. There was no difference detected in the four signaling pathways between the E13.5 wild type and *Osr2-Cre* ^*KI*^ *; Rosa26R-Fgf8* anterior palatal shelves (data not shown). However, an elevation of Phosphorylated Erk (pErk) was detected in the medial side of *Osr2-Cre* ^*KI*^ *; Rosa26R-Fgf8* posterior palatal shelves, but not in the wild type posterior palatal shelves (Fig 6A, 6A’, 6B and 6B’). For the Jak-Stat pathway, the level of phosphorylated Jak1 (pJak1) was drastically up-regulated in the mutant palatal mesenchyme compared with the wild type control (Fig 6C, 6C’, 6D and 6D’), while the phosphorylated Jak2 (pJak2), which is restricted in the wild type lateral palatal mesenchyme, diminished from the mutant palatal mesenchyme, but was drastically up-regulated in the mutant epithelium (Fig 6E, 6E’, 6F and 6F’). In contrast, the level of the phosphorylated PLCγ1 (pPLCγ1) and the phosphorylated Akt (pAkt) exhibited no discrepancy in the palatal mesenchyme between the *Osr2-Cre* ^*KI*^ *; Rosa26R-Fgf8* mice and their wild type littermates (Fig 6G–6J). These alterations on FGF signaling pathways suggested that the ectopically activated FGF8 stimulated mesenchymal cell proliferation indiscriminately in all of the mesenchyme through pJak1/Stat pathway and specifically in medial mesenchyme through pErk pathway. Moreover, the cell proliferation of palatal epithelium could be disabled by pJak2-Stat pathway.

## Discussion

### Ectopic activation of *Fgf8* results in increased cell proliferation and failure of elevation of palatal shelves

Cleft palate is one of the most common congenital defects in human newborns. The high morbidity is attributed to the susceptibility of the finely regulated events to various influences during palatogenesis. The mouse models mimicking human cleft palate revealed that cell proliferation is the most subtle event to be affected. Most cases of cleft palate resulted from a reduced cell proliferation, which retarded outgrowth from maxillary process and horizontal growth toward mid-line of palatal shelves [7, 10, 11, 30, 35, 36]. The hypothesis that cell proliferation is involved in the re-orientation and elevation of palatal shelves has been reviewed in detail [1]. In our study, the mesenchymal cell proliferation drastically increased in both the medial and lateral sides by the over-expressed *Fgf8* in mesenchyme, which in turn, altered the palate size and morphology, but cleft palate still happened due to the failure of elevation of the palatal shelves, indicating that the sole horizontal expansion of palatal shelves is insufficient to complete the palatal elevation. During normal palatogenesis, palate elevation is initiated from the anterior part in a “flip-up” manner and extends to posterior palate [1]. In the *Osr2-cre* ^*KI*^;*Rosa26R-Fgf8* palate, although the elevation has been initiated in the anterior, it failed in the posterior half. Therefore, the ectopic FGF8 not only enhances mesenchymal cell proliferation, but also impairs the reorientation of the posterior palatal shelves.

During palatogenesis, *Fgf7* and *Fgf10* are confined to the medial and lateral mesenchyme, respectively. *Fgf7* knockout mouse survives to adulthood without cleft palate [37]. Even in the *Dlx5* null mice, in which *Fgf7* expression is greatly decreased, the palatal shelves are able to elevate to horizontal position [16]. By contrast, the palatal shelves of *Fgf10* deficient mouse stay vertical at E15 with a reduced size [8]. Therefore, although the ectopic activation of *Fgf8* suppressed the expression of *Dlx5* and *Fgf7*, the ablation of *Fgf10* expression in the developing palate is regarded as the cause of the failure of palatal elevation.

### Reverse effects of ectopic FGF8 on the cell proliferation of palatal epithelium and mesenchyme

In normal palate development, FGFR2b is distributed in the epithelium and FGFR2c in mesenchyme [8]. FGF10 signals epithelial cells by binding the FGFR2b to maintain the epithelial cell proliferation [7]. Previous studies showed that FGFR2c had a much higher affinity to FGF8 binding than FGFR2b, while FGF10 had the highest affinity to FGFR2b [38]. Therefore, we assume that the ectopic activated *Fgf8* directly enhanced both the lateral and medial mesenchymal proliferation through FGFR2c. Previous genetic screening demonstrates that the mutations of *Fgfr1* and *Fgfr3* are also associated with human cleft palate [39, 40]. Recently, ablation of *Fgfr1* from neural crest cells was reported to compromise the cell proliferation, elevation and fusion of palate [41]. These results suggest that FGF8 can stimulate the cell proliferation through FGFRs, especially FGFR1, more than FGFR2c. On the other hand, the reduced cell proliferation in palatal epithelium is attributed to the diminished *Fgf10* expression in *Osr2-cre* ^*KI*^;*Rosa26R-Fgf8* palate, instead of the direct inhibition by FGF8, because ectopically activated *Fgf8* in palatal epithelium has no effect on epithelial proliferation and *Shh* transcription. Based on the unaffected epithelial cell proliferation, the normal *Shh* expression and the elevation of palate, it is suggested that the FGF8 did not disrupt the *Fgf10-Shh* interaction loop in *Shh-cre*;*Rosa26R-Fgf8* palate. Interestingly, cell proliferation was decreased in both the medial and lateral mesenchyme of *Shh-cre*;*Rosa26R-Fgf8* palate. The controversial effect on cell proliferation by FGF8 may result from the earlier activation of *Osr2-cre* than *Shh-cre* [20, 23], and /or the different dosage of FGF8 produced by mesenchyme and epithelium, considering the larger amount of mesenchymal cells than that of epithelial cells.

### Differential FGF signaling pathways are involved in the cell proliferation and *Fgf10* expression in palatal shelves

In the *Fgfr2* ^*C342Y/C342Y*^ mutants, the transcription of intracellular FGF signaling pathway suppressors, *Sprouty2* and *Sprouty4* is up-regulated [39, 40]. Deficiency of *Sprouty2* increases mesenchymal cell proliferation and the level of pErk in the developing palate [42]. Therefore, we speculate that the imbalanced cell proliferation, delayed elevation and reduced size of palatal shelves in the *Fgfr2* ^*C342Y/C342Y*^ mutants result from the up-regulated Erk signaling pathway. Our study found that the up-regulated pErk was concentrated in the *Osr2-cre* ^*KI*^;*Rosa26R-Fgf8* medial mesenchyme, instead of the entire palatal mesenchyme. Combined with the ablation of *Dlx5* and *Fgf7* in the same region, we assume that FGF/pErk signaling pathway promotes the cell proliferation, but is unable to maintain *Fgf10* expression in the medial mesenchyme. Meanwhile, the down-regulated pJak2 in the *Osr2-cre* ^*KI*^;*Rosa26R-Fgf8* lateral mesenchyme might also be involved in the loss of *Fgf10* transcription. Similarly, the elevated pJak1 level throughout the palatal mesenchyme may be regarded as the activator for mesenchymal cell proliferation.

### The altered gene expression along the mediolateral axis of the developing palate shelves

In the E13.5 *Osr2-cre* ^*KI*^;*Rosa26R-Fgf8* palate, the enlarged medial and lateral mesenchyme are accompanied with the diminished *Dlx5* and *Fgf7* expression, and the disappeared *Fgf10* expression, respectively. The *Osr2-*expressing domain was also reduced along lateral mesenchyme. Although these alterations suggest the attenuation of lateral identity of *Osr2-cre* ^*KI*^;*Rosa26R-Fgf8* palate, it is hard to conclude if the mediolateral patterning of the *Osr2-cre* ^*KI*^;*Rosa26R-Fgf8* palatal shelves has been impaired because of the lack of a functional marker identifying the medial or lateral mesenchyme of palate. For this reason, whether the fate of the medial and lateral mesenchyme is changed by the ectopic FGF8, still requires further investigation. Although *Wnt5a* plays an essential role in the cell migration along the anterior-posterior axis [4], its unaffected pattern in *Osr2-cre* ^*KI*^;*Rosa26R-Fgf8* palate suggests that *Wnt5a* is not involved in mediolateral identity.

Walker and Fraser hypothesized that horizontal outgrowth of the medial mesenchyme remodeled the vertical posterior palatal shelves into elevating shelves [1]. The horizontal outgrowth is at least partially mimicked by increased medial cell proliferation in the *Osr2-cre* ^*KI*^;*Rosa26R-Fgf8* palate. As demonstrated in our study, the excessive proliferation in medial mesenchyme by over-expressed *Fgf8* failed to compensate for the failure of elevation. Considering the alteration on the gene expression along the mediolateral axis of palatal shelves, the mediolateral patterning may be impaired. The cleft palate caused by loss of mediolateral patterning most likely results from disrupted cell migration. This proposal is supported by the decreased glycosaminoglycan accumulation and synthesis in the gain-of-function of *Fgfr2* mutation [39]. Although the existence of cell migration along the mediolateral axis during palate elevation is still in debate, the cell migration along the mediolateral axis is the one of the most preferred events for further investigation. Furthermore, there is no impact on FGF signaling pathway in the *Osr2-Cre* ^*KI*^ *; Rosa26R-Fgf8* anterior palate, implying that the responsiveness to FGF signaling also varies along the A-P axis of palatal shelves. Further study on the intracellular FGF signaling pathway that regulates gene expression and cell behavior will provide us a thorough understanding of its role in the palatogenesis.
